# Nationwide analysis on sex differences in diagnosis, treatment and survival of rectal cancer

**DOI:** 10.1186/s13293-026-00863-3

**Published:** 2026-02-22

**Authors:** D. M. Mens, V. M. T. van Verschuer, J. M. van Rees, R. R. J. Coebergh van den Braak, C. Verhoef, D. E. Hilling

**Affiliations:** 1 Department of Surgical Oncology and Gastrointestinal Surgery, Erasmus MC Cancer Institute, University Medical Center Rotterdam, PO Box 2040, Rotterdam, 3000 CA The Netherlands; 2 Department of Hepato-Pancreato-Biliary- and Transplant Surgery, University Medical Center Rotterdam, Rotterdam, The Netherlands; 3 Department of Surgery, Catharina Hospital, Eindhoven, The Netherlands

## Abstract

**Objective:**

Limited literature is available comparing sexes in rectal cancer. This nationwide study using real-world data was performed to evaluate sex-based differences in diagnosis, treatment and survival outcomes in rectal cancer.

**Methods:**

Data from the Netherlands Cancer Registry were analyzed for patients diagnosed with rectal adenocarcinoma between 2015 and 2019. Patient and tumor characteristics, treatment strategies, response to neoadjuvant therapy, and survival outcomes were compared between sexes.

**Results:**

The cohort consisted of 22251 patients (37.1% women, 62.9% men). Women more frequently presented with cT4 tumors (16% vs. 11%, P < 0.001) but no differences were observed in nodal status, distant metastases, use of neoadjuvant (chemo) radiotherapy and radicality in resections between sexes. In the total study population, 5-year survival did not differ significantly (63.6% in women vs. 61.6% in men, P=0.23). However, in surgically treated patients, survival was higher in women (77.4% vs. 75.0%, P=0.019). Female sex was an independent predictor for survival in surgically treated patients (HR 0.90; 95% CI 0.82–0.98). In the subgroup of patients who were asymptomatic at the time of diagnosis [n=1320], there were no sex-based differences in presentation, treatment, or survival (5-year overall survival: 78.8% vs. 80.4%, P=0.45).

**Conclusion:**

Sex-based differences exist in rectal cancer presentation and outcome. Women are more likely to present a more advanced T-stage. Despite this, women have a better overall survival after surgical treatment. In contrast, men and women undergoing treatment for asymptomatic rectal cancer have comparable outcomes.

**Supplementary Information:**

The online version contains supplementary material available at 10.1186/s13293-026-00863-3.

## Plain language summary

Rectal cancer affects both men and women, but it is unclear whether the disease develops or progresses differently between the sexes. Understanding these differences could help improve diagnosis, treatment, and outcomes for all patients.

In this nationwide study from the Netherlands, we analyzed information from over 22,000 people diagnosed with rectal cancer between 2015 and 2019. About one-third of the patients were women. We found that women were more likely than men to have locally advanced tumors at diagnosis. Despite this, men and women received similar treatments, including surgery, radiation, and chemotherapy.

Overall, long-term survival was similar for men and women. However, among patients who underwent surgery, women lived slightly longer than men. When we looked specifically at patients whose cancer was detected before they developed symptoms, for example, through national screening programs, there were no differences between men and women in how the cancer presented, how it was treated, or how long they lived.

These results suggest that women are diagnosed with more advanced rectal tumors but still have slightly better survival after surgery. The findings also highlight the importance of early detection of rectal cancer, as patients diagnosed before symptoms appear have much higher survival rates. Understanding sex-based differences in rectal cancer may help refine screening strategies and improve care for both men and women.

**Supplementary Information:**

The online version contains supplementary material available at 10.1186/s13293-026-00863-3.

## Highlights

Tumor presentation: Women were more likely to present with advanced-stage tumors (cT4), with 16% of women diagnosed with cT4 compared to 11% of men. However, no significant differences in nodal or metastatic status were observed between sexes

No treatment differences: There were no significant sex-based differences in treatment strategies, including rates of surgical treatment, neoadjuvant chemoradiotherapy, and radical resections.

Survival Outcomes: Overall, women had a slight survival advantage after surgical treatment, with a 5-year survival of 77.4% compared to 75.0% in men (P=0.019). Female sex was an independent predictor for better survival in surgically treated patients

Asymptomatic patients: Among asymptomatic patients, there were no significant differences between men and women.

Importance of early detection: The study highlighted the critical role of early detection in improving survival outcomes, as asymptomatic patients had significantly better survival than symptomatic ones, regardless of sex

**Supplementary Information:**

The online version contains supplementary material available at 10.1186/s13293-026-00863-3.

## Introduction

Sex differences in disease presentation, treatment and outcomes are increasingly gaining attention especially in fields such as cardiology and psychiatry [1, 2]. However, data on the impact of sex differences in oncological practice are limited.

For colorectal cancer, several studies have reported higher odds on delayed diagnosis or advanced disease in female patients [3–6]. Despite these findings, studies specifically that focus on rectal cancer are scarce. Current treatment protocols for colorectal cancer do not differ between men and women, although the distinct differences between male and female pelvic anatomy may lead to differences in symptom presentation and treatment. A Spanish study showed that women with screen-detected rectal cancers more often had stage IV disease at presentation, and were less frequently treated with neoadjuvant therapy. However, the study did not provide data on pT-stage, treatment and treatment response [7]. Anatomical differences between men and women such as a wider pelvis in women, might contribute to delayed symptom onset, later involvement of the mesorectal fascia (MRF +) and/or surrounding pelvic organs and consequently a lower likelihood of receiving neoadjuvant chemoradiation. Additionally, rectal cancer diagnosis may be delayed in women [7], because the symptoms at presentation may be mistaken for gynecological conditions or pelvic organ prolaps.

The aim of this study is to explore the role of sex on disease presentation, neoadjuvant treatment response and as a potential prognostic factor for survival in patients with rectal cancer, including a subgroup analysis of asymptomatic presentation.

## Methods

### Data collection

In the Netherlands, data of primary rectal cancer patients are registered in the Netherlands Cancer Registry (NCR), using the Dutch Pathological-Anatomical National Automated Archive (PALGA) and the National Registry of Hospital Discharge Diagnoses as data sources. Patient and tumor characteristics were extracted from medical records by trained data clerks. Data on vital status were acquired by linking the NCR to the National Municipal Personal Records Database, which is updated annually, with the latest update on April 1 st, 2024.

Data from all patients with rectal cancer diagnosed between 2015–2019 were extracted from the NCR. Tumor topography and morphology were registered according to the International Classification of Diseases for Oncology (ICD-O). Patients with adenocarcinomas located in the rectum were included in the analyses. Stage of disease was noted according to the TNM classification, using the latest available edition at time of diagnosis. The definition of tumor stage was by clinical (c)TNM, complemented with pathological (p)TNM when available. In patients who did not undergo resection or who only received neoadjuvant chemotherapy or chemoradiotherapy cTNM was used. According to Dutch national guidelines for colorectal cancer, preoperative radiotherapy typically consists of short-course radiotherapy (25 Gy/5 fractions) or neoadjuvant chemoradiotherapy (50–50.4 Gy/25–28 fractions with concomitant capecitabine) [8]. Neoadjuvant treatment response was identified as tumor regression after neoadjuvant chemoradiotherapy according to the Mandard tumor regression (TRG) score [9].

### Statistical analyses

Data were presented as count with percentages or median with interquartile ranges (IQR). Categorical data of male versus female patients were compared using the Chi Square test. Continuous data were analyzed using the Mann Whitney U test. Tumor regression was categorized into two categories: pathological complete response (TRG 1) and incomplete response (TRG 2–5). Survival was presented in months and defined as time from diagnosis until death; patients who were alive at last follow up were censored. Survival data were presented using the Kaplan–Meier method and the log rank test was used to assess survival differences. Covariates of influence were identified on survival using Cox’s proportional hazard analysis. Variables with a p-value of < 0.10 in univariate analyses or clinically relevant were entered in the multivariate model. A p-value of < 0.05 was considered statistically significant. All statistical analysis were performed using SPSS (version 28.0, IBM Inc, Chicago, IL) and R version 4.0.2 (R Project for Statistical Computing, Vienna, Austria).

A subgroup analysis was performed on patients diagnosed in 2015 to assess sex differences in asymptomatic patients, as 2015 was the only year with a high level of data completeness for symptom status. Asymptomatic presentation was classified as diagnosis through screening, surveillance, incidental findings or other reasons, while symptomatic presentation was categorized as diagnosis due to complaints.

## Results

**Fig. 1:**
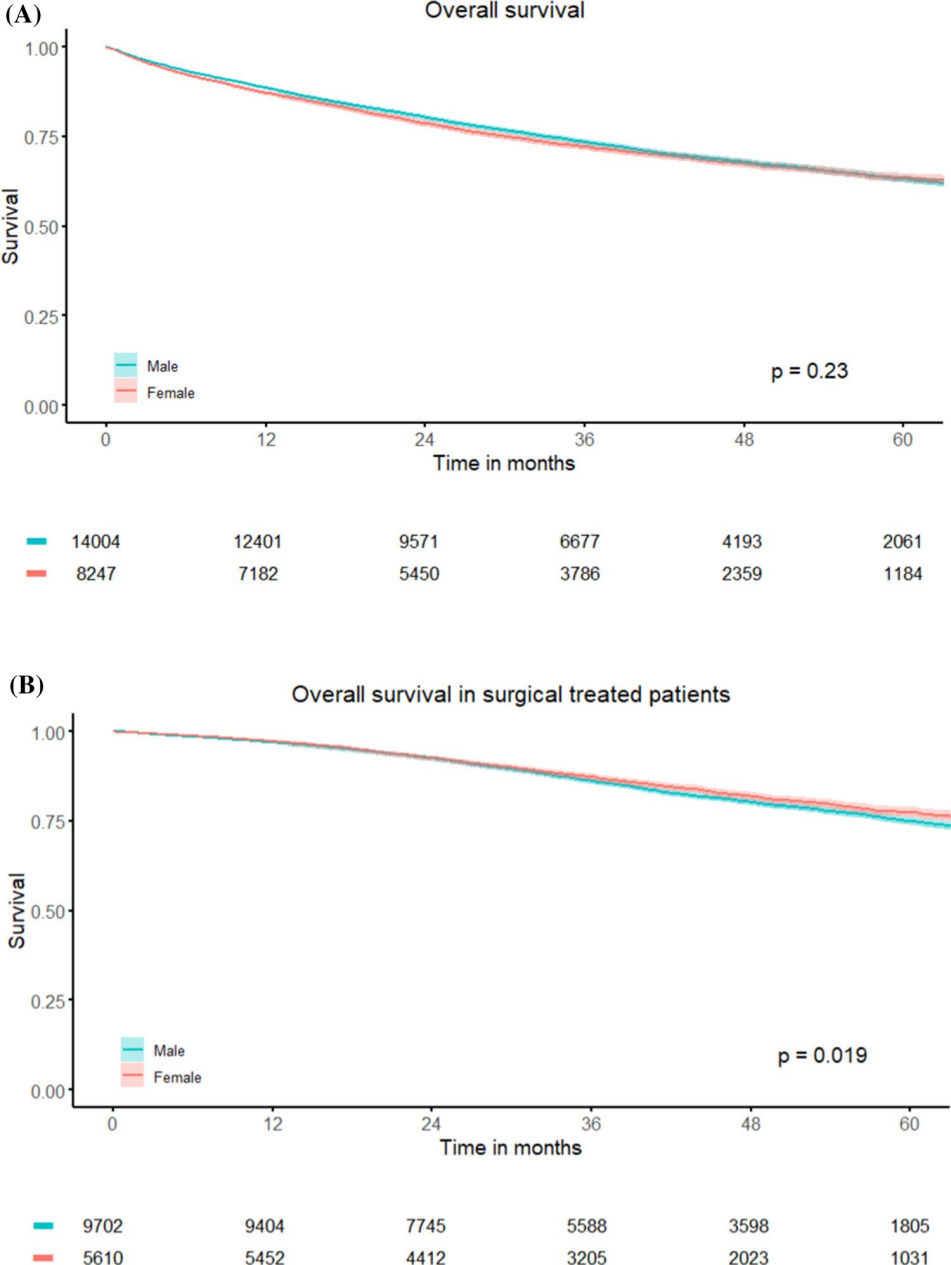
**A**. Survival of male versus female rectal cancer patients. **B**. Survival differences between male versus female rectal cancer patients in surgical treated patients

In the Netherlands from 2015 to 2019, 14004 men (62.9%) and 8247 women (37.1%) were diagnosed with adenocarcinoma of the rectum (Table 1). Women presented relatively more often with cT4 cancers (16% [n = 1164]) compared to men (11% [n = 1325]; P < 0.001), and accordingly, 329 (6%) of women had p/ypT4 cancer vs. 366 (4%) of men (P < 0.001). There were no differences in nodal status or metastatic status at presentation. Of all patients, 94.5% received a form of cancer-directed treatment, i.e. (chemo-)radiotherapy, surgery or (palliative) chemotherapy. Rates of surgical treatment were similar between sexes (68% of women [n = 5610] and 69% of men [n = 9702]; P = 0.051), as were the rates of preoperative radiotherapy (34% of both men and women; P = 0.961) and preoperative chemoradiotherapy (20% [n = 1670] of women and 19% of men [n = 2701], P = 0.08). There were no differences in radical resection rates (95% of women [n = 5173] and 95% of men [n = 8892]; P = 0.160). After neoadjuvant chemoradiotherapy, there was no significant difference in complete response rate between sexes (P = 0.08); (Supplemental Table 1). Survival analysis showed no difference in overall survival between men and women for all T-stages (5-year OS: 61.6% in men vs. 63.6% in women, P = 0.23) nor for cT4 specifically (5-year OS: 37.1% in men vs. 40.2% in women, P = 0.083) (Fig. 1A and Supplemental Fig. 1). However, among all surgical treated patients, women had significantly better survival (5-year OS: 75.0% in men vs. 77.4% in women, P = 0.019) (Fig. 1B).

**Table 1:** Baseline characteristics of male and female patients with rectal cancer diagnosed between 2015–2019

	level	Male	Female	p	Missing (%)
n		14004	8247		
Age (median [IQR])		68.0 [61.0, 75.0]	68.0 [59.0, 76.0]	0.593	0.0
cT (%)	T1	1355 (11)	808 (11)	** < 0.001**	10.7
	T2	2797 (22)	1625 (22)		
	T3	7077 (56)	3711 (51)		
	T4	1325 (11)	1164 (16)		
cN (%)	N0	6649 (50)	3849 (49)	0.543	4.8
	N1	3708 (28)	2146 (27)		
	N2	3012 (23)	1810 (23)		
cM (%)	-	11,701 (84)	6902 (84)	0.769	0.0
	M1	2302 (16)	1343 (16)		
p/ypT (%)	T1	2568 (26)	1515 (26)	** < 0.001**	29.0
	T2	3011 (30)	1691 (29)		
	T3	4083 (41)	2246 (39)		
	T4	366 (4)	329 (6)		
p/ypN (%)	N0	5852 (67)	3397 (66)	0.267	37.4
	N1	2104 (24)	1287 (25)		
	N2	835 (9)	462 (9)		
Distance to anorectal junction (%)	0—5.0 cm	3714 (52)	2155 (50)	0.562	48.4
	> 5.0—10.0 cm	2224 (31)	1357 (32)		
	> 10.0—15.0 cm	1032 (14)	630 (15)		
	> 15.0 cm	223 (3)	142 (3)		
Presentation	complaints (%)	3592 (66)	2226 (70)	** < 0.001**	61.3
	screening (%)	1561 (29)	798 (25)	** < 0.001**	61.3
	surveillance (%)	74 (1)	48 (2)	0.558	61.3
	incidental (%)	132 (2)	58 (2)	0.070	61.3
	other (%)	53 (1)	26 (1)	0.471	61.3
	unknown (%)	26 (0)	11 (0)	0.371	61.3
Tumor-targeted therapy (%)		13,297 (95)	7735 (94)	** < 0.001**	0.0
Neoadjuvant radiotherapy (%)		4754 (34)	2797 (34)	0.96	0.0
Neoadjuvant chemoradiotherapy (%)		2701 (19)	1669 (20)	0.08	0.0
Surgery (%)		9702 (69)	5610 (68)	0.051	0.0
Tumor regression (%)	Complete regression	551 (18)	372 (20)	0.070	77.8
	Incomplete regression	2525 (82)	1489 (80)		
Radical resection (%)		8892 (94)	5173 (95)	0.160	32.9
MRF status (%)	MRF +	3028 (60)	1931 (68)	** < 0.001**	64.8
MSI status (%)	MSI	118 (2)	80 (2)	0.354	58.7

In patients who underwent surgery, independent predictors for superior overall survival were female sex (HR 0.90; 95% CI 0.82–0.98), neoadjuvant chemoradiotherapy (HR 0.82; 95% CI 0.74–0.91) and a radical resection (HR 0.39; 95% CI 0.35–0.44). Age (HR 1.05; 95% CI 1.04–1.05), cT3-4 carcinoma (vs. cT1-2; HR 1.62; 95% CI 1.45–1.80), cN2 status (vs. cN0 status HR 1.28; 95% CI 1.13–1.45) and cM1 status (vs. cM0 status HR 3.54; 95% CI 3.16–3.95) were associated with poorer overall survival (Supplemental Table 2).

**Table 2:** Asymptomatic rectal cancer patients 2015

	level	Male	Female	p	Missing (%)
n		890	430		
Age (median [IQR])		67 [63, 69]	66 [63, 69]	0.481	0.0
cT (%)	T1	130 (17)	62 (17)	0.092	15.3
	T2	230 (30)	126 (35)		
	T3	375 (49)	151 (42)		
	T4	25 (3)	19 (5)		
cN (%)	N0	516 (62)	253 (63)	0.686	6.8
	N1	206 (25)	89 (22)		
	N2	107 (13)	59 (15)		
cM (%)	-	833 (94)	396 (92)	0.313	0.0
	M1	57 (6)	34 (8)		
p/ypT (%)	T1	294 (40)	140 (40)	0.809	17.3
	T2	234 (32)	105 (30)		
	T3	210 (28)	108 (31)		
	T4	0 (0)	0 (0)		
p/ypN (%)	N0	422 (70)	213 (71)	0.689	31.8
	N1	141 (24)	65 (22)		
	N2	37 (6)	22 (7)		
Distance to anorectal junction (%)	0—5.0 cm	245 (30)	109 (28)	0.639	8.0
	> 5.0—10.0 cm	343 (42)	160 (41)		
	> 10.0—15.0 cm	220 (27)	106 (27)		
	> 15.0 cm	18 (2)	13 (3)		
Presentation	screening (%)	765 (86)	362 (84)	0.181	0.0
	surveillance (%)	33 (4)	27 (6)		
	incidental (%)	82 (9)	35 (8)		
	other (%)	10 (1)	6 (1)		
Tumor-targeted therapy (%)	Yes	874 (98)	425 (99)	0.388	0.0
Neoadjuvant radiotherapy (%)	Yes	149 (17)	67 (16)	0.523	0.0
Neoadjuvant chemoradiotherapy (%)	Yes	176 (20)	95 (22)	0.329	0.0
Tumor regression (%)	Complete	31 (20)	18 (24)	0.427	52.6
	Incomplete	126 (80)	56 (76)		
Surgery (%)	Yes	688 (77)	339 (79)	0.530	0.0
Radical resection (%)	Yes	648 (95)	320 (95)	0.544	2.5
MSI status (%)	Yes	3 (3)	1 (2)	0.726	87.2

### Asymptomatic rectal cancer

In the full cohort from 2015–2019, women more often presented with complaints than men (70% of women [n = 2226] vs. 66% of men [n = 3592]; P < 0.001), and less often with screen-detected cancers (25% of women [n = 798] vs. 29% of men [1561]; P < 0.001) (Table 1).

**Fig. 2:**
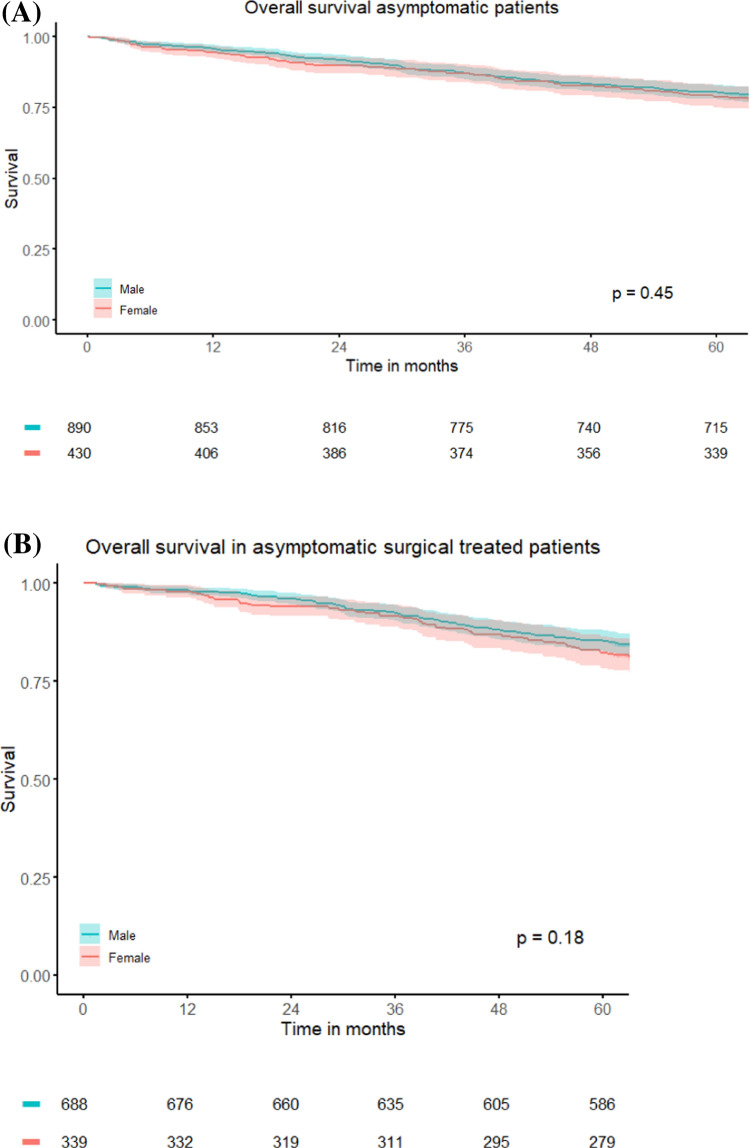
**A**. Survival differences male versus female patients within the asymptomatic group. **B**. Survival differences between male versus female surgically treated asymptomatic rectal cancer patients

In the subgroup of patients diagnosed with rectal cancer in 2015, data on clinical presentation (symptomatic or asymptomatic) was available in 4746 patients (98.2%). A minority were asymptomatic at presentation (n = 1320, 27.8%). Baseline characteristics of these patients are shown in Table 2. No differences in clinical presentation, clinical/pathological tumor stage, neoadjuvant treatment and tumor regression after neoadjuvant treatment, or surgical uptake were observed between male and female patients. No significant differences were shown in overall survival between male and female patients in all asymptomatic patients (5-year OS 80.4% in men vs. 78.8% in women, P = 0.45) nor in asymptomatic surgically treated patients (5-year OS 85.3% in men vs. 82.3% in women, P = 0.18) (Fig. 2A and 2B).

Similar, no significant difference in 5-year OS was observed between male and female patients who presented with symptoms (54.5% vs. 57.0%, P = 0.25) (Supplemental Fig. 2). However, a large difference in 5-year OS was observed between asymptomatic and symptomatic patients (79.9% and 55.5%, P < 0.0001, respectively) (Supplemental Fig. 3).

## Discussion

This nationwide, real-world data study showed an sex-based overview of rectal cancer incidence and outcomes in the Netherlands between 2015–2019. Altough female patients presented more frequently with locally advanced tumors, particulary cT4 tumors, univariate analysis did not demonstrate a significant difference in overall survival between sexes. However, multivariate analysis among surgically treated patients identified female sex as an independent predictor of improved survival, alongside younger age, lower nodal status, absence of distant metastases at diagnosis, neoadjuvant chemoradiotherapy, and radical resection.

Our findings indicate that approximately 4500 patients are diagnosed with rectal cancer annually, with a higher incidence observed in male patients compared to females. In line with previous studies, the incidence of rectal cancer is higher in male patients [10, 11]. The underlying reasons may include biological factors such as hormone levels, as high oestrogen levels are associated with an decreased risk of developing colorectal cancer as well as dietary and lifestyle factors [12]. The higher prevalence of cT4 tumors in women raises the question of whether this is attributable to tumor biology, suggesting more aggressive disease in females, diagnostic delay in acknowledging symptoms in female patients, or potential inefficacies in the national screening program. In the current study, no significant sex-based differences were observed in asymptomatic patients suggesting that the disparity in cT4 tumor prevalence is unlikely to result from screening performance. This reinforces the need to explore sex-specific tumor biology, anatomic differences and diagnostic delay as potential contributing factors.

This study found no significant sex-based differences in treatment strategies, including surgical intervention, neoadjuvant therapy, and radical resection rates, indicating that treatment decisions in rectal cancer are not influenced by patient sex. Nevertheless, female patients who underwent surgery demonstrated superior survival outcomes compared to males, findings that are in line with existing literature [13, 14]. This survival advantage may reflect differences in postoperative recovery or biological factors, such as immunological or hormonal differences, but research on these topics is lacking. Differences in surgical complexity may also contribute to the observed survival advantage in women. Female patients may experience less complex surgeries due to anatomical factors, such as a wider pelvis, which could make the surgical approach less challenging and potentially reduce complications, leading to higher survival rates.

Previous research by *Sarasqueta *et al*.* reported a higher incidence of stage IV rectal cancer in screen-detected female patients [7]. However, our study did not find a significantly higher proportion of stage IV asymptomatic female rectal cancer patients at presentation. The increased detection of cT4/p/ypT4 tumors in the entire group (symptomatic and asymptomatic) female patients may be explained by misinterpretation of early symptoms as non-malignant conditions. This is further supported by our finding that asymptomatic patients did not show a higher prevalence of cT4/p/ypT4 rectal tumors, so sex-based differences were only observed in symptomatic patients. Additionally, no significant sex-based differences were observed in the occurrence of distant metastases at diagnosis in asymptomatic patients, suggesting that female patients are not more likely to be diagnosed with stage IV disease via screening. Interestingly, while female sex was an independent predictor of superior survival in the entire surgically treated cohort, no such benefit was seen among surgically treated asymptomatic patients. This discrepancy may be explained by unmeasured confounders or the smaller data availability in this subgroup, which was restricted to solely 2015.

Contrary to *Sarasqueta *et al*.,* who reported that female patients were less likely to receive neoadjuvant (chemo)radiotherapy [7], our study found no such sex-based disparity. The discrepancy between our studies may be attributed to the larger sample size in our cohort. Also, variations in colorectal cancer screening protocols between Spain and the Netherlands may contribute to differences in diagnostic stage and treatment outcomes. The Spanish CRC screening program invites individuals aged 50–69 years to submit faeces samples for immunochemical fecal occult blood testing (iFOBT), with a compliance rate of approximately 55% [15]. In contrast, the Dutch national screening program targets individuals aged 55–75 years and achieves a higher compliance rate of 70–75% for participation in the iFBOT [16]. These differences highlight how national screening strategies and health system structure might influence diagnostic stage and treatment patterns, potentially affecting sex-related outcomes [17]. A notable finding in our study is the significantly higher 5-year survival in asymptomatic patients compared to symptomatic patients, emphasizing the critical role of early detection in rectal cancer in improving outcomes, irrespective of sex.

Our results are further supported by the population-based study by *van Erning *et al., which analyzed sex differences in colorectal cancer over a 10-year period. In their rectal cancer subgroup (which did not distuighuish between symptomatic and asymptomatic cases), no significant sex-based disparities were observed in stage at presentation or treatment allocation. Notably, older women (≥ 70 years) were more likely than men to receive neoadjuvant therapy in stage II–III disease, and survival outcomes were generally comparable between sexes. Only in women aged 56–70 years with stage IV rectal cancer a modest but statistically significant excess mortality was observed [18]. These findings align with our results, reinforcing that, within the Dutch healthcare context, treatment decisions are primarily driven by clinical criteria rather than sex.

The strengths of this study include its large-scale, long-term, nationwide dataset, which provides a representative overview of sex-related differences in rectal cancer incidence, treatment and survival. Furthermore, the inclusion of a large asymptomatic cohort adds important nuance that has been underreported in existing literature. However, its retrospective design introduces inherent limitations, including potential confounding factors. Additionally, the NCR lacks detailed molecular and genetic data, limiting our ability to analyze tumor biology as a factor in survival outcomes. Furthermore, the observed 2.4% difference in 5-year OS between men and women in surgically treated patients, while statistically significant, is relatively small in clinical terms. Additionally, the 3.1% difference in survival for cT4 tumors did not reach statistical significance (P = 0.083), suggesting that the sample size may not have provided sufficient statistical power to detect a significant difference. This raises the possibility of Type 2 errors, where true differences might not be detected due to insufficient power, especially in subgroups with smaller sample sizes.

In conclusion, while previous reports have suggested sex-based differences in rectal cancer presentation and survival, our study show largely comparable results between men and women, except for a small but significant advantage in surgically treated female patients. These findings underscore the importance of early detection and emphasize that standardized, evidence-based treatment protocols can help ensure optimal outcomes for all rectal cancer patients, regardless of sex.

## Supplementary information

Additional file 1.

Additional file 2.

Additional file 3.

Additional file 4.

Additional file 5.

## Data Availability

All data was retrieved from the open-access Netherlands Cancer Registry. All data related to the research is available upon reasonable request.
